# Mitigation of Hepatic and Pancreatic Injury by *Spondias tuberosa* Extract Through Oxidative Enzyme Modulation and TGF‐β Downregulation

**DOI:** 10.1002/fsn3.71846

**Published:** 2026-07-03

**Authors:** Vitória Natália Ferreira de Sena, Cecília Paulino Cassiano da Silva, Letícia Alves Borges e Pires, Ludmila Thainá Chaves Freitas, Raimundo Fernandes de Araújo Júnior, Bento João Abreu, Islania Giselia Albuquerque Araujo, Isac Almeida de Medeiros, Bruno Raniere Lins de Albuquerque Meireles, Rafael Andre da Silva, Diego Dias dos Santos, Cristiane Damas Gil, Karina Carla de Paula Medeiros

**Affiliations:** ^1^ Department of Morphology Federal University of Rio Grande do Norte Natal Rio Grande do Norte Brazil; ^2^ Department of Health Sciences Federal University of Paraíba João Pessoa Paraíba Brazil; ^3^ Center of Agro‐Food Science and Technology Federal University of Campina Grande Campina Grande Paraíba Brazil; ^4^ Department of Morphology and Genetics Federal University of São Paulo São Paulo São Paulo Brazil

**Keywords:** experimental diabetes mellitus, morphology, *Spondias tuberosa*

## Abstract

Diabetes mellitus (DM) is a chronic metabolic disorder marked by hyperglycemia due to impaired insulin secretion and/or action. Conventional therapies are often limited by side effects, highlighting the need for alternative treatments. Natural products have gained interest as promising therapeutic agents. *Spondias tuberosa* Arruda (umbu), traditionally used in Brazilian folk medicine, exhibits pharmacological potential against metabolic diseases. This study evaluated organoprotective and antioxidant effects of *S. tuberosa* extract (ExSt) in streptozotocin (STZ)‐induced diabetic rats through clinical, biochemical, histopathological evaluations, as well as analysis of oxidative stress‐related markers. Fifty‐two Wistar rats were divided into five groups: CT (nondiabetic control), DM (diabetic), EX (nondiabetic + ExSt), DMEX (diabetic + ExSt), and DMIN (diabetic + insulin). Diabetes was induced by STZ (50 mg/kg, i.p.), and treatments with ExSt (500 mg/kg, orally) or NPH insulin (10 IU, s.c.) were administered for 30 days. Diabetic rats exhibited hyperglycemia, polyphagia, polydipsia, weight loss, and increased transaminases (AST, ALT), along with hepatic and pancreatic alterations. Treatment with ExSt moderately reduced AST and ALT levels, strongly preserved pancreatic morphology, and attenuated hepatic glycogen depletion by 50% despite the maintenance of hyperglycemia. Additionally, ExSt downregulated TGF‐β1 and TGF‐β2 expression and modulated oxidative stress‐related enzymes, indicating antioxidant and antifibrotic mechanisms. These findings demonstrate that *S. tuberosa* extract mitigates hepatic and pancreatic injury in experimental diabetes through modulation of oxidative enzymes and TGF‐β signaling, supporting its potential as a natural therapeutic alternative derived from Brazil's biodiversity.

## Graphical abstract

Treatment with *Spondias tuberosa* extract ameliorated pancreatic and hepatic morphological damage in STZ‐induced diabetic rats, potentially by reducing oxidative stress and modulating profibrotic signaling mediated by TGF‐β.

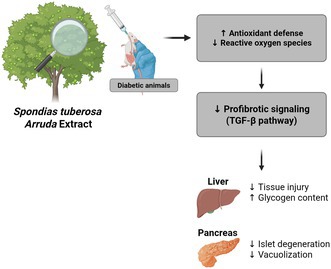

## 1 Introduction

Diabetes mellitus (DM) is one of the most prevalent endocrine‐metabolic disorders worldwide, characterized by persistent hyperglycemia resulting from impaired insulin production by pancreatic β‐cells or defects in insulin sensitivity in peripheral tissues. Chronic metabolic dysregulation contributes to progressive microvascular and macrovascular complications, making DM a major global cause of morbidity and mortality (Okur et al. 2017).

Epidemiological projections show a worrying upward trend. The International Diabetes Federation estimated that 537 million adults were living with DM in 2021, and this number continues to rise. In Brazil, recent data from the Brazilian Diabetes Society suggest that by 2040, approximately 1.8 million individuals will be affected by type 1 diabetes (T1DM), with up to 0.5 million deaths attributable to the disease (International Diabetes Federation 2025; de Pittito et al. 2023). Such estimates highlight the growing public health burden associated with DM.

Although the etiology of DM is complex and multifactorial, genetic and environmental factors are known to interact in triggering the autoimmune destruction of β‐cells observed in T1DM (Luo et al. 2023). Furthermore, recent research trends have highlighted the role of environmental pollutants, such as microplastics, which may contribute to pancreatic dysfunction and exacerbate metabolic damage (Huang et al. 2022; Mierzejewski et al. 2025). Sustained hyperglycemia leads to systemic toxicity and triggers progressive cellular dysfunction in multiple organs, including the liver (Gupta et al. 2024).

The liver is central to glucose homeostasis through regulation of glycogen synthesis and hepatic gluconeogenesis. Under diabetic conditions, however, hepatocytes become vulnerable to metabolic overload, oxidative stress, and inflammation. Consequently, hepatic complications such as steatosis, necrosis, and hemorrhage may develop over time (Mohamed et al. 2016; Sulaiman 2019). The resulting overproduction of reactive oxygen species (ROS) promotes lipid peroxidation, membrane instability, and progressive parenchymal damage.

Although standard antidiabetic medications effectively control glucose levels, long‐term therapy is frequently accompanied by adverse effects such as gastrointestinal disturbances, hepatotoxicity, heart failure, and weight gain (Chaudhury et al. 2017; Marín‐Peñalver et al. 2016; Russell‐Jones and Khan 2007; Widyawati et al. 2015). Thus, the search for safer and more effective alternatives remains a crucial clinical and scientific priority. Natural products have emerged as valuable sources of new therapeutic agents due to their bioactive properties and potential for reduced side effects (Governa et al. 2018).

*Spondias tuberosa* Arruda (Anacardiaceae), commonly known as umbu or umbuzeiro, is an endemic species of the Brazilian Caatinga biome and widely used in traditional medicine for the treatment of DM and other inflammatory and infectious conditions (de Albuquerque et al. 2007; Barbosa et al. 2018; Siqueira et al. 2016). Previous studies demonstrated antidiabetic activity in extracts obtained from its root bark, supporting the potential pharmacological value of this species (Barbosa et al. 2018).

Considering this traditional knowledge and earlier evidence, further investigation into other plant parts is warranted. Therefore, this study evaluated the hydroalcoholic extract of the fruit peel of *S. tuberosa* Arruda (ExSt) for its ability to protect the liver and pancreas in a STZ‐induced diabetes model in rats. Biochemical, histopathological, and histochemical analyses were performed to elucidate the extract's potential mechanisms of action and contribute to the development of novel therapeutic strategies for diabetes‐induced organ damage.

## 2 Materials and Methods

### 2.1 Animals

This study was carried out following the Guide for the Care and Use of Laboratory Animals of the National Institutes of Health, approved by the Research Ethics Committee on the Use of Animals of the Federal University of Rio Grande and approved on January 12, 2022, under protocol number 056/2022, in which the animals to be used were provided by the Central Bioterium of the Institute of Biosciences at UFRN. The study involved 52 male Wistar rats ( *Rattus norvegicus* ), 90 days old, weighing 250–300 g. The animals were randomly assigned to five groups: Control group (CTL, *n* = 8), diabetic group (DM, *n* = 12), healthy group treated with *S. tuberosa* extract 500 mg/kg (EX, *n* = 8), diabetic group treated with *S. tuberosa* extract 500 mg/kg (DMEX, *n* = 12), and diabetic group treated with NPH insulin subcutaneously (s.c.) 10 IU once daily (DMIN, *n* = 12). The animals were housed in polypropylene cages equipped with drinking and feeding bottles during a 1‐week acclimatization period. Four animals were allocated per cage, kept under controlled temperature conditions (24°C ± 2°C) and lighting (12‐h light/12‐h dark cycle), with ad libitum access to food and water.

### 2.2 Obtainment of the Lyophilized Hydroalcoholic Extract From the Fruit Peel of *S. tuberosa* Arruda (ExSt)

Fruits of *S. tuberosa* Arruda were collected from a rural property in Olivedos, Paraíba, Brazil, under authorization from SisGen (no. A0BD05B). After selection, fruits showing damage, contamination, or heterogeneous ripening were discarded. The selected fruits were washed, sanitized, and manually peeled; only the peels were used. The peels were stored at −20°C, then frozen at −80°C for 24 h, and dried in a convection oven at 45°C for 48 h. The dried material was ground using a commercial mixer, further refined with a mortar and pestle, and sieved (45 mesh). The resulting powder was extracted with a hydroalcoholic solution (ethanol:water 50:50, v/v) at a solid‐to‐solvent ratio of 5% (w/v) using ultrasonic‐assisted extraction (42 kHz, 16 min). Extracts were vacuum filtered (3 μm) to obtain clear hydroalcoholic solutions. Ethanol was removed using a rotary evaporator at 45°C until the volume was reduced by half. The remaining aqueous extracts were frozen at −80°C for 24 h and lyophilized under vacuum (0.024 mBar) for 48 h, yielding the lyophilized hydroalcoholic extract of *S. tuberosa* fruit peels (Ex*St*). The extract was stored at −20°C until further experiments.

### 2.3 Identification and Quantification of Total Phenolic Compounds in the ExSt by High‐Performance Liquid Chromatography (HPLC)

Chromatographic analyses of the ExSt at a concentration of 5 mg/mL were performed using a high‐performance liquid chromatography (HPLC) system from Shimadzu (Kyoto, Japan), equipped with a Rheodyne 7125i automatic injector and a UV/VIS detector. The columns used were a Shimadzu LC‐18 column (25 cm × 4.6 mm, 5 μm particle size; Supelco, Bellefonte, PA, USA) and a Shimadzu C‐18 ODS guard column.

For the identification of phenolic compounds, the samples were eluted using a gradient system composed of solvent A (2% acetic acid, v/v) and solvent B (acetonitrile: methanol, 2:1, v/v) as the mobile phase, with a flow rate of 1 mL/min. The column temperature was maintained at 25°C, and the injection volume was 20 μL.

The gradient elution program was as follows: 90% A at 0 min, 80% A at 10 min, 70% A at 15 min, 60% A at 25 min, 50% A from 30 to 40 min, 75% A at 42 min, and 90% A at 44 min. Phenolic compound peaks were monitored at 280 nm. The LabSolutions software (Shimadzu) was used to control the LC‐UV system and for data processing (Alcântara et al. 2019).

### 2.4 Experimental Design

**FIGURE 1:**
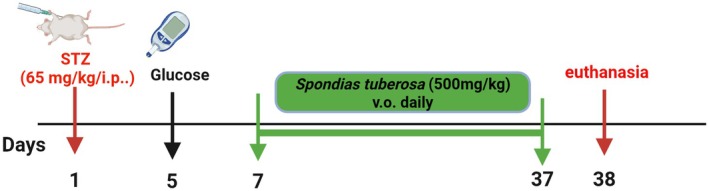
Experimental design.

To establish the experimental model of type 1 diabetes mellitus (T1DM), an intraperitoneal (i.p.) injection of STZ (50 mg/kg) dissolved in sodium citrate solution (pH 4.5) at 10 mmol/L was administered (de Carvalho et al. 2003). On the fifth day after diabetes induction, blood glucose levels of the groups were measured using a portable glucometer (ONETOUCH, Ultra), and animals with blood glucose ≥ 250 mg/dL were considered diabetic. Treatments for the EX, DMEX, and DMIN groups started 1 week after diabetes induction, by oral gavage (v.o.) and lasted for 30 consecutive days. After 30 days, 24 h following the last treatment, the animals were anesthetized with 2% xylazine (10 mg/kg) and 10% ketamine (75 mg/kg) for blood collection by cardiac puncture, and then euthanized for sample removal (Figure 1).

### 2.5 Biochemical and Clinical Analyses

Each rat was weighed individually before the start of the experiment (initial weight) and regularly throughout the experimental period until the end of the experiment (final weight). Water consumption was measured using a graduated cylinder with the provided amount (500 mL), and the amount not consumed was subtracted, while food consumption was measured daily for each group using a digital scale (Balmak ELCN‐15). The provided amount (300 g) was weighed, and the amount not consumed was subtracted, obtaining the daily consumption value. The blood of the euthanized animals was collected through cardiac puncture. The samples were placed in test tubes and centrifuged for 15 min (1800 rpm). The serum obtained was collected and sent for analysis at the Onofre Lopes University Hospital (HUOL), where biochemical analysis was performed using the automated equipment Wiener Lab CMD 800iX1 (Rosario, Argentina).

### 2.6 Histological and Histomorphometrical Procedures

The organs were removed from the animal after euthanasia, weighed, and fixed in buffered formalin solution (10%) for 24 h. Subsequently, the tissue samples followed the preestablished histological processing steps. The sections were stained with hematoxylin and eosin (HE) and periodic acid‐Schiff (PAS). For the morphometric analysis of the pancreas, five fields of the organ were selected on each slide for photo capture at ×200 and ×400 magnification using an optical microscope (OLYMPUS, CX21) coupled with a digital camera. The area measurement and the quantification of the number of vacuoles in each islet were performed using the ImageJ program. In the morphometric analysis of hepatic tissue, stained with PAS, intracellular glycogen was evaluated using light microscopy and photographed in five fields at ×400 magnification on the same microscope coupled with a digital camera. Finally, the quantitative analysis was performed with the assistance of the ImageJ program.

### 2.7 Immunohistochemistry

Sections were deparaffinized using two types of xylene solution, hydrated by ethanol in various gradients, and antigen retrieval was obtained by quenching with 3% sodium citrate (pH 6.4) for 15 min at 90°C. Sections were then washed with PBS and incubated with primary anti‐superoxide dismutase‐1 (SOD‐1) antibodies (Cat# G0214, 1:400; Santa Cruz Biotechnology) overnight in a humid chamber at 4°C. Sections were then incubated with secondary anti‐rabbit peroxidase antibody (Cat# BA1054‐0.5, 1:25; Boster Biological Technology) also at 4°C. Subsequently, immunoreactivity to the protein was observed using the colorimetry‐based detection kit following the protocol provided by the manufacturer. Nuclear counterstaining was performed with Mayer's hematoxylin for 2 min. The stained sections were examined using a Nikon DS‐Fi2 optical microscope (Nikon Corporation, Tokyo, Japan, Department of Morphology/UFRN) at ×40 objective. The intensity of immunostaining was scored as follows: 0 = absence of positive cells, 1 = a small number of positive cells or isolated cells, 2 = moderate number of positive cells, and 3 = a large number of positive cells. The score was determined by multiplying the percentage of stained cells and the intensity (de Carvalho et al. 2023). The analysis was performed in a double‐blind manner by two previously trained examiners.

### 2.8 TGF‐β Measurement

The levels of TGF‐β isoforms were quantified in liver tissue homogenates, using the Milliplex MAP Rat Cytokine/Chemokine Magnetic Bead Panel and its protocol (Merck, Darmstadt, Germany). The samples were then analyzed on the Luminex 100/200, and data were collected using the Luminex xPO‐NENT 3.1 software (Luminex, Santa Clara, USA).

### 2.9 ROS Measurements

Redox‐sensitive fluorescent dye (DHE) was used to evaluate ROS (reactive oxygen species) formation. The liver was isolated and embedded in an OCT compound, and transferred and stored in a freezer at −80°C until the next step of experimentation. Microtomy of the tissue in cryostat was performed at −20°C, in which cuts with 5 μM thickness were obtained. The tissue was fixed on slides, washed with phosphate‐saline buffer (PBS) (161.0 mM NaCl; 1.8 mM NaH_2_PO_4_·H_2_O, and 15.8 mM Na_2_HPO_4_), and incubated with DHE (5 μM) for 30 min, at 37°C, in a humid chamber protected from light (Widyawati et al. 2015). Subsequently, the sections were washed (twice) before being mounted in Fluorescence Mounting Medium (DAKO) with coverslips. Images were obtained with a Fluorescence Eclipse Ti‐U Nikon microscope (Japan). Quantification (of levels of staining) was performed using ImageJ software. The data were normalized using the CTL group and expressed as percentage fluorescence.

### 2.10 Activity of Oxidative and Antioxidant Enzymes

The levels of oxidative and antioxidant markers, including nitric oxide (NO), superoxide dismutase (SOD), and catalase (CAT), were determined in liver homogenates obtained under different experimental conditions. Briefly, liver tissue samples (0.1 g) were homogenized in 1 mL of assay‐specific homogenization buffer using a BeadBlaster 24 homogenizer (Benchmark Scientific Inc., USA) for 30 s. The homogenates were subsequently centrifuged, and the supernatants were collected for biochemical analyses. Nitric oxide production was indirectly assessed by measuring nitrite levels using the Griess reaction. Equal volumes of sample and Griess reagent were incubated at room temperature, and absorbance was measured at 540 nm using a BioTek 800 TS microplate reader (BioTek Instruments, USA). Nitrite concentrations were calculated from a sodium nitrite standard curve. Superoxide dismutase activity was determined based on the inhibition of pyrogallol autoxidation. Briefly, 30 μL of liver homogenate was mixed with 6 μL of MTT, 15 μL of pyrogallol, and 99 μL of phosphate buffer (pH 8.0). After incubation for 20 min at 40°C, absorbance was measured at 570 nm. Catalase activity was evaluated by monitoring the decomposition rate of hydrogen peroxide (H_2_O_2_). Samples were diluted (1:10) in phosphate buffer (pH 7.0), and the decrease in absorbance was recorded at 375 nm for 3 min. Hydrogen peroxide consumption values were corrected by subtracting the respective blank readings. Total protein concentration in liver homogenates was quantified using the Thermo Scientific Pierce Bradford Protein Assay (Thermo Scientific, USA), according to the manufacturer's instructions, with absorbance measured at 595 nm. All oxidative and antioxidant parameters were normalized to total protein content and expressed as μmol/mg protein.

### 2.11 Statistical Analysis

Data are presented as mean ± standard deviation (SD). Normality was assessed using the Shapiro–Wilk test. Comparisons between experimental groups were performed using one‐way analysis of variance (ANOVA) followed by Tukey's multiple comparisons test, using GraphPad Prism (version 8.0) (GraphPad Software Inc., San Diego CA, USA). The effect size for ANOVA analyses was estimated using the eta‐squared (*η* ^2^) method, calculated as the ratio between the sum of squares of the treatment effect and the total sum of squares. The significance level was set at *p* < 0.05. The number of animals per group (8–12) was determined based on previous experimental studies using rodent models with diabetes and diabetic complications, in which group sizes generally range from 6 to 12 animals, depending on the variability of the measured parameters (Alsuliam et al. 2023; Gulle et al. 2014; Tang et al. 2018). This sample size was considered sufficient to detect biologically relevant differences, respecting the principles of reduction in animal experimentation.

## 3 Results

### 3.1 Phytochemical Composition of ExSt

HPLC of ExSt revealed the presence of 12 phenolic compounds which were expressed in compound mg/100 g ExSt concentrations. The phenolic compounds identified included phenolic acids such as caffeic acid, salicylic acid, and 3,4‐dihydroxybenzoic acid, as well as flavonoids such as myricetin and catechin (Table 1).

**TABLE 1:** Phenolic compounds identified in hydroalcoholic extract from the fruit peel of *Spondias tuberosa* Arruda (5 mg/mL) by HPLC.

Phenolic compounds	Concentration of the compound mg/100 g of ExSt
**Phenolic acids**	
3,4‐Dihydroxybenzoic acid	0.0032
4‐Hydroxybenzoic acid	0.000880
p‐Coumaric acid	0.0002
Salicylic acid	0.0042
Sinapic acid	0.0006
Trans‐cinnamic acid	0.0002
Vanillic acid	0.0024
Ferulic acid	0.0006
Ellagic acid	0.0008
Caffeic acid	0.0042
**Flavonoids**	
Myricetin	0.0032
Catechin	0.0012

### 3.2 Body Weight, Water and Food Intake

Table 2 shows the clinical analysis after 4 weeks of STZ‐induced DM. DM and DMEX animals presented significant body weight loss (*p* < 0.001) and increased water and food intake (*p* < 0.001) compared with controls (CT). The DMIN group improved these parameters (*p* < 0.05), whereas EX animals remained similar to CT.

**TABLE 2:** Body weight (BW), feed consumption (FC), and water intake (WI).

	CT	EX	DM	DMEX	DMIN
Body weight (g)	368 ± 26	369 ± 21	307 ± 43^#^	316 ± 43^#^	462 ± 14*
Feed consumption (g/24 h)	44 ± 4	55 ± 5	84 ± 7^#^	83 ± 8^#^	57 ± 4*
Water intake (mL/24 h)	35 ± 5	28 ± 6	140 ± 24^#^	132 ± 21^#^	55 ± 9*

*Note:* Data are expressed as mean ± SD. Differences were detected by Tukey's test.

Abbreviations: CT, control group; DM, diabetes mellitus group; DMEX, diabetes mellitus group treated with *S. tuberosa* extract; DMIN, diabetes mellitus group treated with insulin; EX, group treated with *Spondias tuberosa* extract.

^#^ *p* < 0.001 versus CT, *p < 0.05 versus DM.

### 3.3 Glucose, Cholesterol, Triglycerides, and Transaminases

**FIGURE 2:**
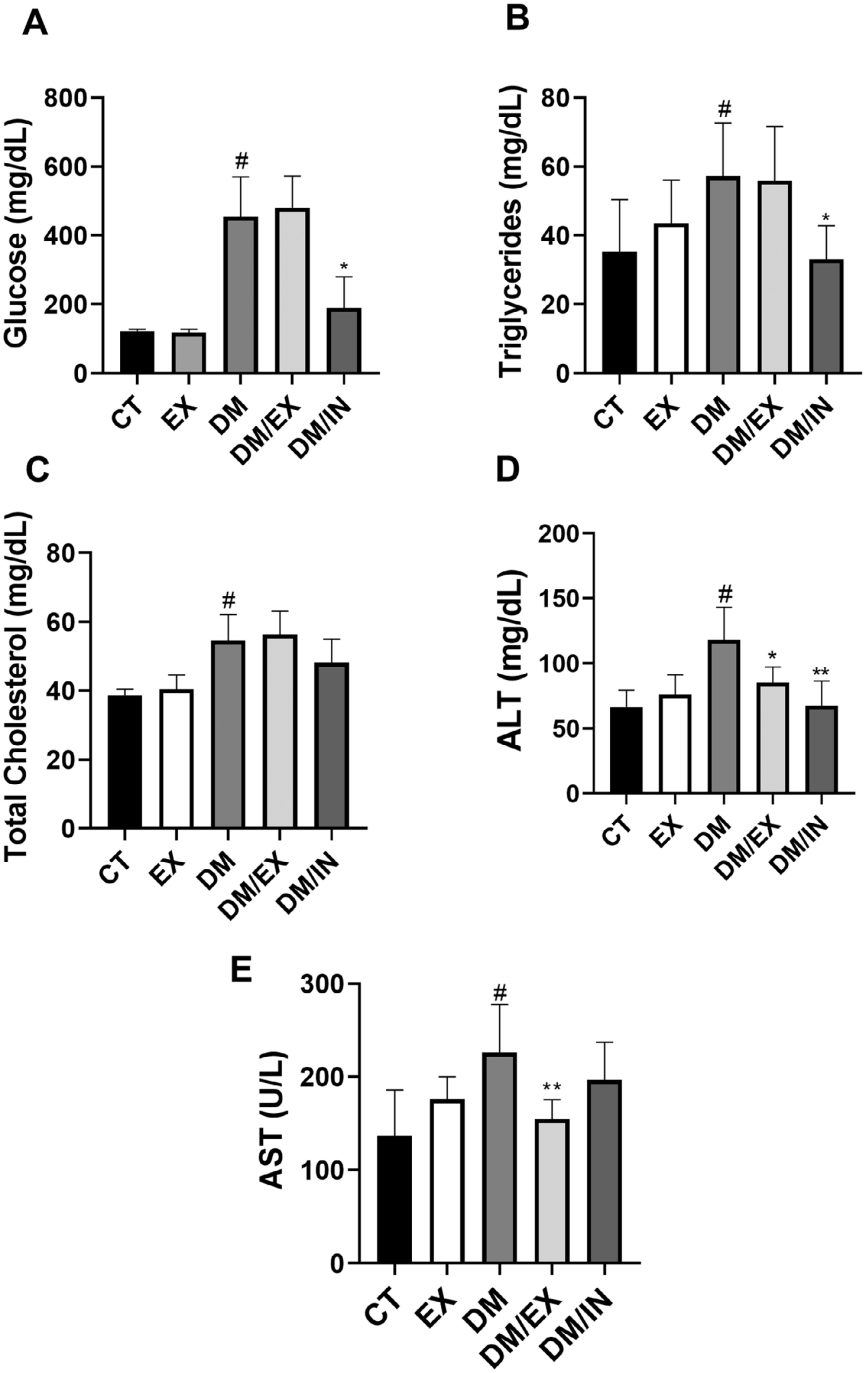
Dosage of glucose (A), triglyceride (B), total cholesterol (C), aspartate aminotransferase (AST) (D), and alanine aminotransferase (ALT) (E) after 30 days of experiment. CT, control group; DM, diabetes mellitus group; DMEX, diabetes mellitus group treated with *Spondias tuberosa* extract; DMIN, diabetes mellitus group treated with insulin; EX, group treated with *S. tuberosa* extract. Data are expressed as mean ± SD. Differences were detected by Tukey's test. ^#^ *p* < 0.001 versus CT, *p < 0.05 versus DM, **p < 0.001 versus DM.

After 4 weeks, DM animals exhibited marked hyperglycemia (~450 mg/dL; *p* < 0.05). Insulin treatment (DMIN) reduced glycemia (~250 mg/dL; *p* < 0.05), while EX animals remained normoglycemic (~150 mg/dL). In contrast, DMEX animals failed to reduce blood glucose (Figure 2A). Regarding the lipid profile, DM animals showed increased triacylglycerides and cholesterol (*p* < 0.05). EX animals were unchanged compared with CT, and DMIN reduced triglycerides (*p* < 0.05), whereas DMEX did not improve lipid parameters (Figure 2B,C). For hepatic enzymes, DM animals exhibited elevated AST and ALT (*p* < 0.01). The DMIN group reduced ALT (*p* < 0.01), while DMEX reduced both AST (*p* < 0.01) and ALT (*p* < 0.05) (Figure 2D,E).

### 3.4 Morphology and Morphometry of Pancreatic Islets

**FIGURE 3:**
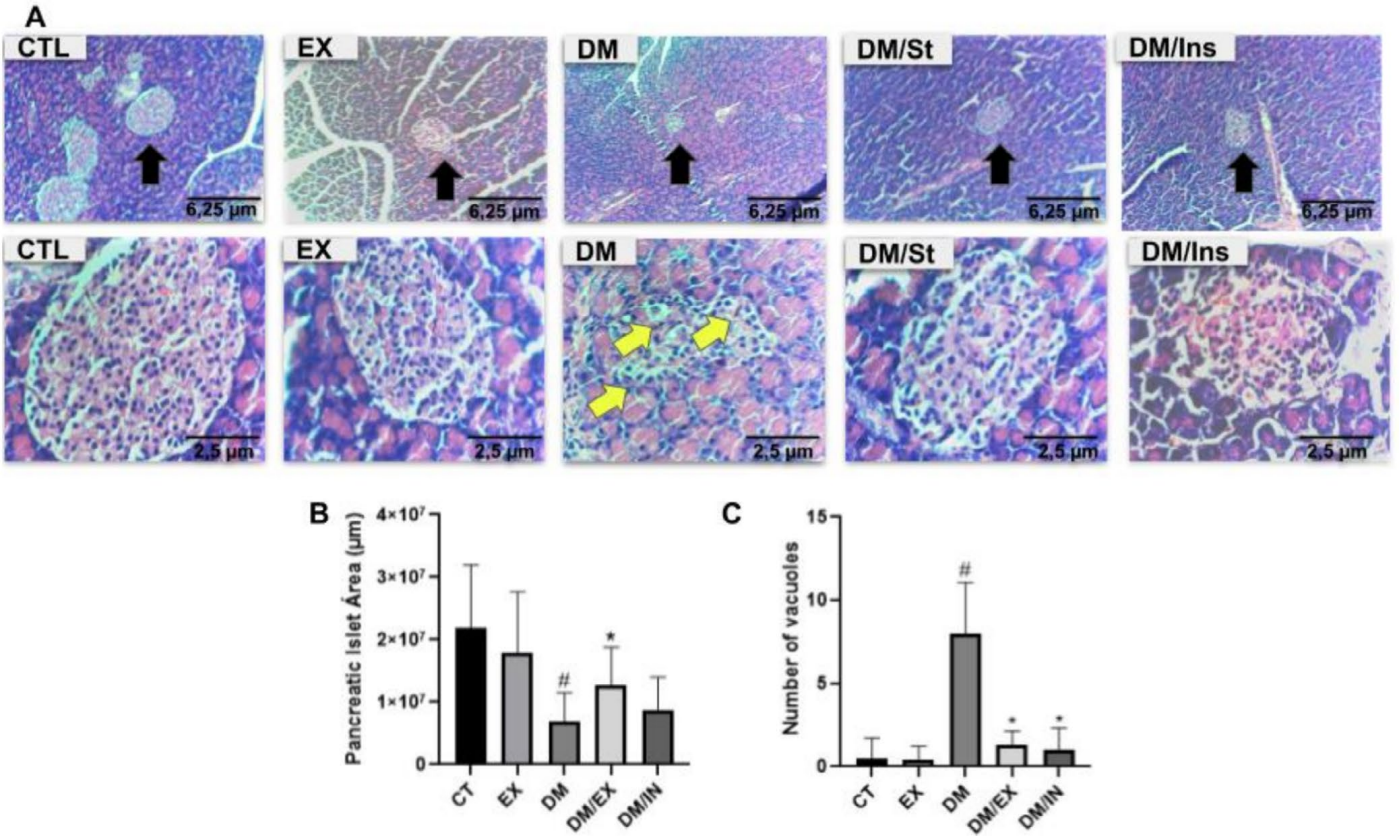
Morphological assessment of the pancreatic islet showing structural integrity in treated groups versus atrophy in DM animals after 30 days of experiment. (A) Photomicrograph of pancreatic tissue. (B) Pancreatic islet area. (C) Number of vacuoles in the pancreatic islet. CT, control group; DM, diabetes mellitus group; DMEX, diabetes mellitus group treated with *Spondias tuberosa* extract; DMIN, diabetes mellitus group treated with insulin; EX, group treated with *S. tuberosa* extract. Scale bar = 2.5 and 6.25 μm (H&E staining, magnification ×100 and ×400). Black arrow—pancreatic islet; yellow arrow—vacuoles in the pancreatic islet. Data are expressed as mean ± SD. Differences were detected by Tukey's test. ^#^ *p* < 0.001 versus CT, *p < 0.05 versus DM.

Representative pancreatic images (Figure 3A) show preserved islet architecture in CT and EX groups, while DM animals displayed irregular contours, atrophy, cell loss, and vacuoles. Partial recovery was observed in DMEX and DMIN groups, with reduced atrophy and fewer vacuoles. Morphometric analysis confirmed decreased islet area in DM animals (Figure 3B, *p* < 0.05), partially restored in DMEX and DMIN, along with significant reduction in vacuole number (Figure 3C, *p* < 0.001).

### 3.5 Morphometric Analysis of Liver Samples Stained With Periodic Acid‐Schiff (PAS)

**FIGURE 4:**
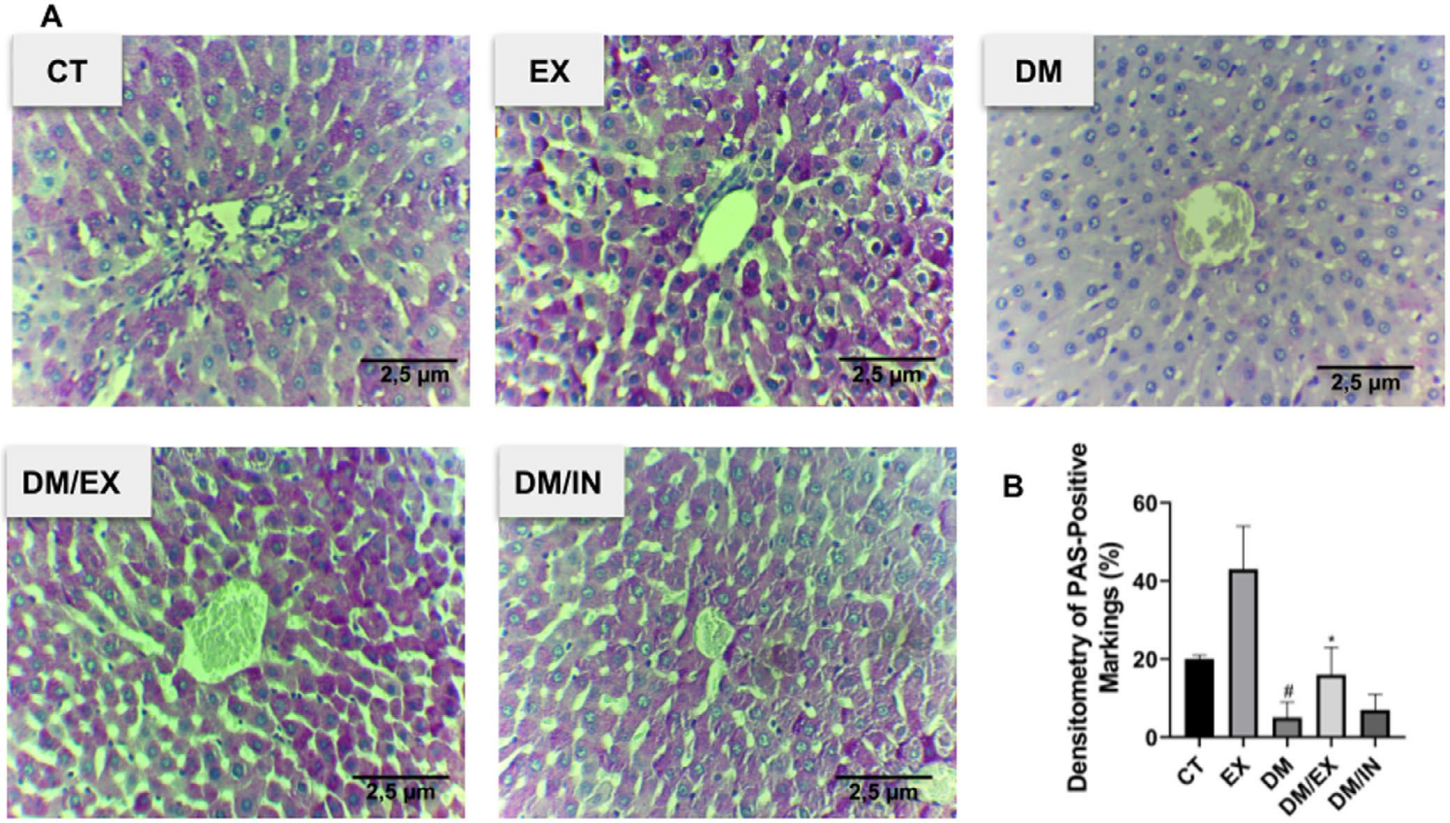
Evaluation of hepatic glycogen depletion after 30 days of experiment. (A) Photomicrograph of liver tissue PAS staining. (B) Densitometry of PAS‐positive markings. CT, control group; DM, diabetes mellitus group; DMEX, diabetes mellitus group treated with *Spondias tuberosa* extract; DMIN, diabetes mellitus group treated with insulin; EX, group treated with *S. tuberosa* extract (PAS staining, ×100 magnification). Scale bar = 6.25 μm. Data are expressed as mean ± SD. Differences were detected by Tukey's test. ^#^ *p* < 0.001 versus CT, *p < 0.05 versus DM.

Figure 4 illustrates the histomorphometric analysis of hepatic tissue stained with PAS, used to identify glycogen deposition or depletion. DM animals exhibited a significant reduction (Figure 4B, *p* < 0.01) in the percentage of PAS‐positive area, indicating glycogen depletion compared with CT animals. DMEX group promoted partial recovery of hepatic glycogen, as demonstrated by an increase in PAS‐positive area (Figure 4B, *p* < 0.05). These histomorphometric numerical data reflect the photomicrographs of hepatic tissue stained with PAS (Figure 4A).

### 3.6 TGF‐β Analysis

**FIGURE 5:**
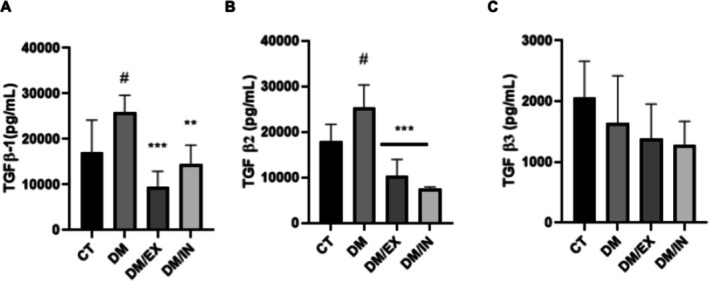
TGF‐β1, TGF‐β2, and TGF‐β3 dosage of liver samples after 30 days of experiment. CT, control group; DM, diabetes mellitus group; DMIN, diabetes mellitus group treated with insulin; DMEX, diabetes mellitus group treated with *S. tuberosa* extract; Data are expressed as mean ± SD. Differences were detected by Tukey's test. ^#^ *p* < 0.001 versus CT, **p < 0.001 versus DM, ***p < 0.0001 versus DM.

Figure 5 shows the levels of the three TGF‐β isoforms in liver tissue homogenates. DM animals exhibited significant increases in TGF‐β1 (Figure 5A, *p* < 0.001) and TGF‐β2 (Figure 5B, *p* < 0.001). Both DMEX and DMIN groups significantly reduced these isoforms (*p* < 0.0001). No differences were observed in TGF‐β3 among the experimental groups (Figure 5C).

### 3.7 Fluorescence Intensity Evaluation

**FIGURE 6:**
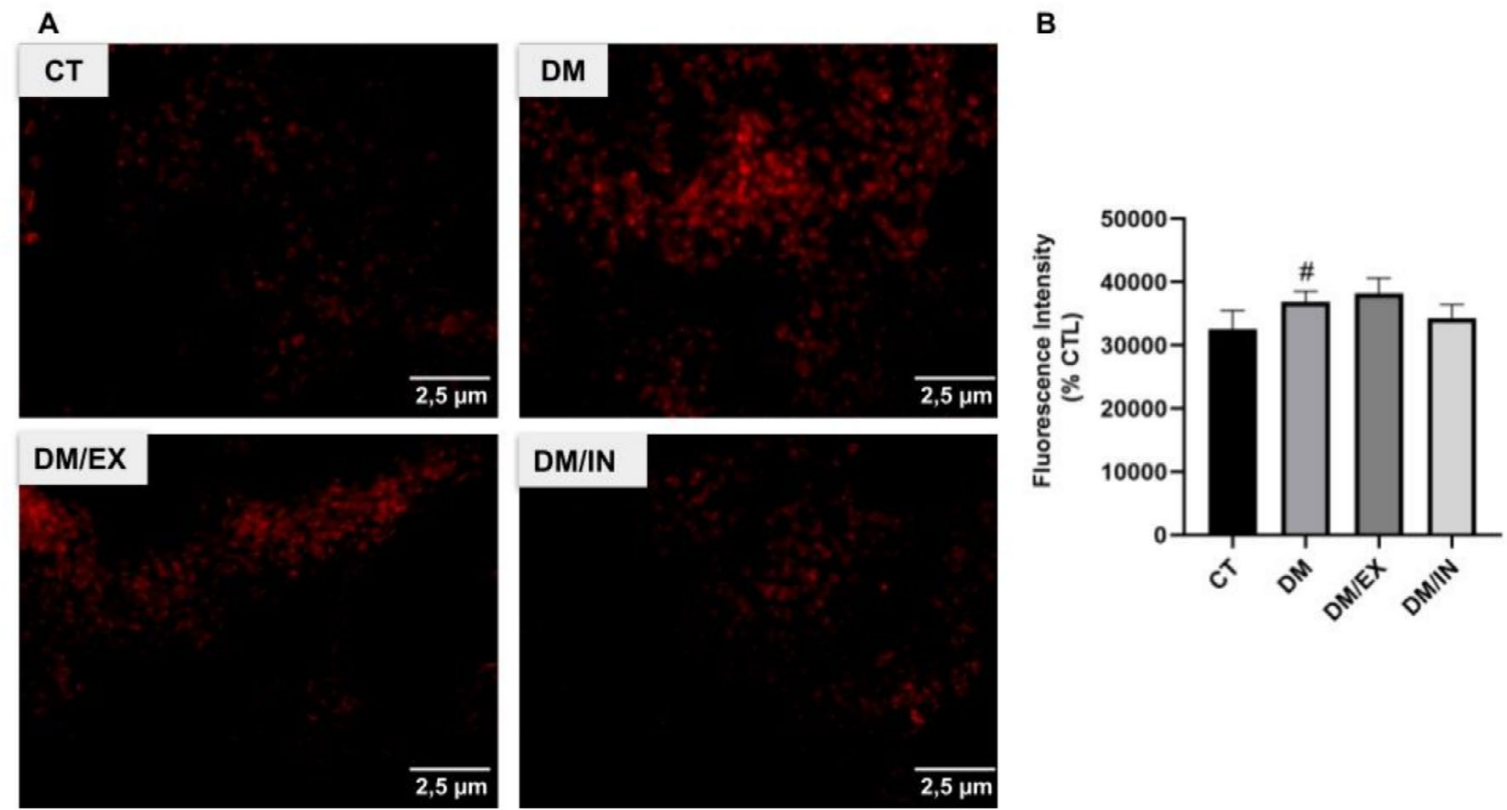
Representative image of liver tissue samples (A) and quantitative analysis (B) of superoxide anion production as measured by fluorescent intensity emitted by the DHE probe, showing persistent ROS levels despite treatment. Liver isolated from experimental groups, treated for 30 days (×200 magnification). CT, control group; DM, diabetes mellitus group; DMEX, diabetes mellitus group treated with *S. tuberosa* extract; DMIN, diabetes mellitus group treated with insulin; EX, group treated with *Spondias tuberosa* extract. Scale bar = 2.5 μm. Data are expressed as mean values of the percentage of fluorescence relative to the control ± SD. Differences were detected by Tukey's test. ^#^ *p* < 0.001 versus CT.

Figure 6 illustrates the fluorescence intensity for superoxide anion in liver tissue homogenates. DM animals showed a significant increase (Figure 6B, *p* < 0.05) compared with CT, while neither DMEX nor DMIN treatments were able to reduce this fluorescence. These histomorphometric data are consistent with the photomicrographic findings of hepatic tissue stained with DHE (Figure 6A).

### 3.8 Evaluation of Enzymatic Activity of Reactive Oxygen Species and SOD Immunohistochemistry

**FIGURE 7:**
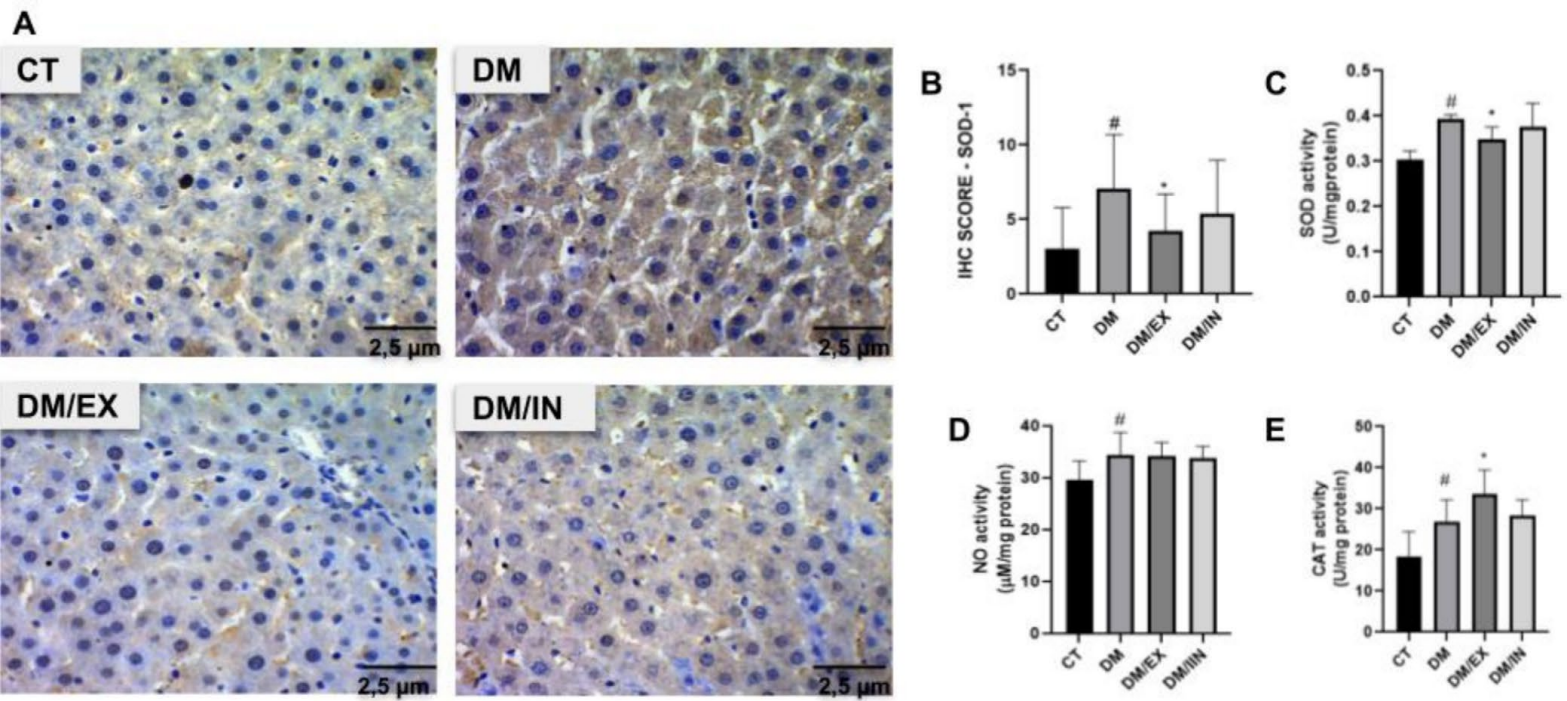
Immunohistochemistry evaluation of superoxide dismutase (SOD) expression and oxidative and antioxidant enzyme activity in liver samples. (A) Representative images of SOD expression in liver tissue. (B) Quantification of SOD expression was obtained by measuring the ratio between DAB and H‐stained areas. (C) SOD, superoxide dismutase activity; (D) NO, nitric oxide activity; (E) CAT catalase activity; CT, control group; DM, diabetes mellitus group; DMEX, diabetes mellitus group treated with *Spondias tuberosa* extract; DMIN, diabetes mellitus group treated with insulin (H & DAB staining, magnification: ×400). Scale bar = 2.5 μm. Data are expressed as mean ± SD. Differences were detected by Tukey's test. ^#^ *p* < 0.001 versus CT, *p < 0.05 versus DM.

Immunohistochemical analysis of hepatic tissue revealed changes in SOD1 expression. Representative images are shown in Figure 7A. Scoring indicated a significant increase in SOD1 immunostaining in DM animals compared with CT (Figure 7B, *p* < 0.001), while DMEX animals showed reduced staining (*p* < 0.05), an effect not observed in DMIN.

The activity of oxidative and antioxidant enzymes in liver tissue was also assessed. DM animals exhibited increased levels of NO (Figure 7C, *p* < 0.001), SOD (Figure 7D, *p* < 0.001), and CAT (Figure 7E, *p* < 0.01) compared with CT. Treatment with *S. tuberosa* extract (DMEX) reduced SOD levels (*p* < 0.05) and enhanced CAT activity (*p* < 0.05) compared with DM, whereas DMIN showed no significant changes.

## 4 Discussion

In this study, the effects of the hydroalcoholic extract of *S. tuberosa* Arruda fruit peel (ExSt) were evaluated on clinical, biochemical, histological, and molecular parameters in a murine model of STZ‐induced diabetes mellitus (DM). The findings revealed that, although ExSt did not significantly reduce blood glucose levels, it exerted notable hepatoprotective, antioxidant, and immunomodulatory effects.

DM, particularly when triggered by β‐cytotoxic agents such as STZ, promotes a metabolic stress environment characterized by persistent hyperglycemia, dyslipidemia, systemic inflammation, and oxidative stress (American Diabetes Association Professional Practice Committee 2024; Barbosa et al. 2018). Collectively, these factors impair the functional integrity of target organs such as the liver and pancreas, contributing to chronic diabetic complications (Governa et al. 2018; Okur et al. 2017).

Although this study utilizes a T1DM model (characterized by absolute insulin deficiency), the findings have implications for human type 2 diabetes (T2DM). In T2DM, chronic oxidative stress and TGF‐β activation are also central to the development of nonalcoholic fatty liver disease (NAFLD) and pancreatic exhaustion (Targher et al. 2018). The ability of ExSt to decouple organoprotection from strict glycemic control suggests it could serve as a complementary therapy in T2DM patients who struggle with glucose variability.

In the present study, STZ‐induced DM resulted in typical clinical alterations—weight loss, polydipsia, and polyphagia—along with pronounced hyperglycemia, reflecting a severe metabolic imbalance and energetic catabolism associated with diabetogenic conditions (Barbosa et al. 2018). ExSt treatment was not able to normalize hyperglycemia or the clinical parameters evaluated. This suggests that ExSt does not directly modulate glycoregulation, unlike other hypoglycemic phytotherapeutics (Barbosa et al. 2018; Giacco and Brownlee 2010).

A study by Barbosa et al. (2018) investigating the root bark of *S. tuberosa* demonstrated glycemic restoration, a discrepancy that may be attributed to the different plant parts used and their distinct phytochemical profiles. Insulin therapy partially reversed the metabolic alterations, in agreement with Petersen and Shulman (2018), who reported its efficacy in restoring metabolic homeostasis.

When interpreting these results, it is essential to acknowledge the limitations of the STZ‐induced model. While STZ is a gold standard for mimicking T1DM due to its β‐cytotoxic action, it is known for nonspecific systemic toxicity, including potential nephrotoxicity, which may confound metabolic observations (Ghasemi et al. 2014). Regarding the treatment, the dose of 500 mg/kg was selected based on previous studies showing its efficacy in other plant parts. Furthermore, previous toxicological assessments of *S. tuberosa* bark have indicated a high safety margin (LD50 > 2000 mg/kg), supporting the use of this concentration without significant acute adverse effects (Barbosa et al. 2016). In the present study, diabetic animals exhibited glucose levels above 250 mg/dL, confirming successful DM induction. This hyperglycemic state was accompanied by elevated AST and ALT levels, indicating metabolic dysfunction and potential hepatic injury, findings consistent with the pathophysiology of DM in which insulin deficiency disrupts energy metabolism and promotes hepatic oxidative stress (Barbosa et al. 2016; Giacco and Brownlee 2010). The liver plays a central role in glucose homeostasis by regulating both storage and release of glucose according to metabolic demand. Accordingly, the marked reduction in hepatic glycogen observed by periodic acid–Schiff (PAS) staining in diabetic animals reflects impaired glucose utilization and metabolic imbalance, in agreement with previous reports (Fan et al. 2020; Mohamed et al. 2016).

Although ExSt treatment did not significantly improve glycemic or lipid profiles, it significantly reduced AST and ALT levels and attenuated hepatic glycogen depletion, suggesting a hepatoprotective effect. The biological effects of ExSt can be attributed to its rich phytochemical profile. Our HPLC analysis identified 12 phenolic compounds, with highlights on caffeic acid and myricetin. Caffeic acid is recognized for its ability to modulate antioxidant enzymes and inhibit inflammatory pathways (Wan et al. 2021), while myricetin has been shown to protect pancreatic β‐cells and improve hepatic function under glucose‐induced stress (Karunakaran et al. 2019). The synergistic action of these polyphenols likely explains the reduction in AST/ALT levels and the preservation of pancreatic islets even in the presence of persistent hyperglycemia (Alam et al. 2021).

Hepatic alterations in DM are directly related to β‐cell degeneration and the consequent impairment of insulin secretion, since the pancreas acts synergistically with the liver in the regulation of glucose metabolism. Pancreatic β‐cell dysfunction represents a central event in the disease and is characterized by cytoplasmic vacuolization (Horii et al. 2022; Yagihashi 2017) and islet atrophy (Ghasemi et al. 2014). In the present experimental model, STZ administration produced pronounced morphological alterations in pancreatic islets, including atrophy and cell death, confirming the effectiveness of diabetes induction. Despite the severity of tissue injury, ExSt treatment attenuated these alterations, suggesting a protective and potentially regenerative effect, similar in magnitude to that observed with insulin therapy.

Based on these findings, it can be inferred that ExSt exerted protective effects on both hepatic and pancreatic tissues, even without fully restoring glycemia. This suggests that its mechanism of action diverges from insulin and likely involves the reduction of oxidative stress and the modulation of inflammatory pathways. Similar mechanisms have been proposed by Clemente‐Suárez et al. (2023), who investigated plant extracts with hypoglycemic and antioxidant properties in experimental models.

Oxidative stress is a key factor in DM pathogenesis and its complications (Baynes 1991; Evans et al. 2002; Robertson et al. 2003). The imbalance between reactive oxygen species (ROS) and antioxidant mechanisms results in cellular damage, inflammation, and endothelial dysfunction. In experimental DM, excessive ROS production has been associated with mitochondrial dysfunction, activation of inflammatory signaling, and induction of apoptosis (Giacco and Brownlee 2010; Rains and Jain 2011). Chronic hyperglycemia enhances mitochondrial ROS formation, activates inflammatory pathways, and promotes apoptotic cell death (Rains and Jain 2011; Robertson et al. 2003).

In this study, diabetic animals exhibited elevated hepatic nitric oxide (NO) levels and increased activities of SOD and CAT, indicating an adaptive response to excessive ROS. ExSt treatment significantly reduced NO levels and partially restored SOD activity, whereas CAT activity was further enhanced compared to the diabetic group, suggesting an additional stimulus to endogenous antioxidant defenses.

A notable finding was that ExSt treatment did not significantly reduce the fluorescence intensity of DHE, which marks superoxide anion production. This indicates that the extract's mechanism may not involve direct scavenging of all superoxide species. Instead, the organoprotection observed appears to be driven by a specific modulation of the enzymatic antioxidant system—particularly the enhancement of catalase (CAT) activity—and the significant downregulation of profibrotic TGF‐β1 and TGF‐β2 isoforms. This suggests a targeted response against hydrogen peroxide‐induced damage and fibrogenesis rather than a generalized reduction of superoxide anions.

Multiple studies highlight that redox imbalance contributes to the activation of cytokines involved in chronic inflammation and fibrogenesis. Among these, transforming growth factor‐β (TGF‐β) is a multifunctional cytokine whose overexpression is promoted by hyperglycemia and oxidative stress (Pacher et al. 2007; Schuster and Krieglstein 2002). TGF‐β regulates inflammatory responses, fibrosis, and tissue remodeling. In our study, diabetic animals showed a significant upregulation of TGF‐β1 and TGF‐β2 in liver tissue homogenates, whereas ExSt treatment markedly reduced both isoforms. These findings may be related to the presence of phenolic compounds, which modulate TGF‐β expression and profibrotic pathways (Ashrafizadeh et al. 2020; El‐Hawary et al. 2019; Yang et al. 2020).

## 5 Conclusion

**FIGURE 8:**
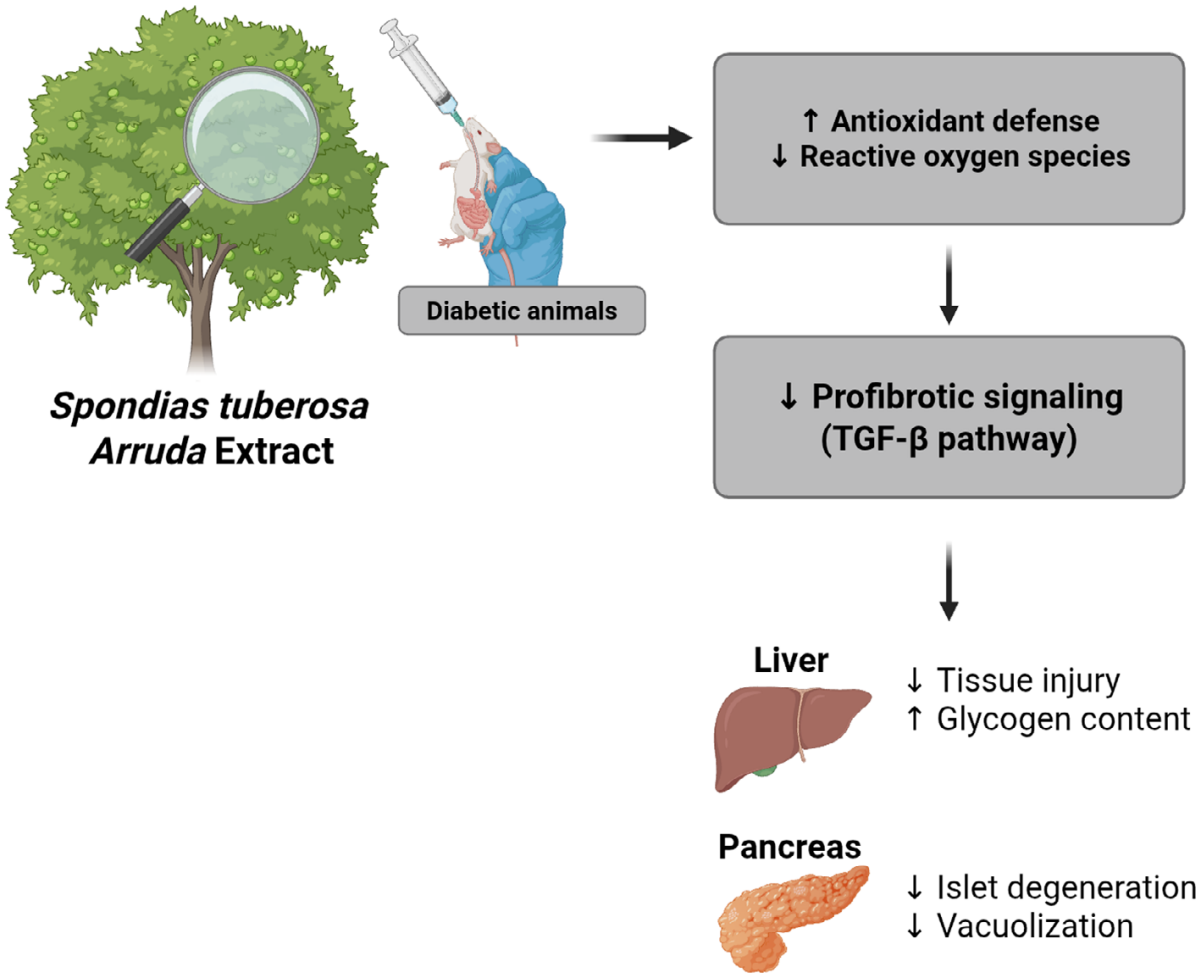
Action of ExSt in an experimental streptozotocin‐induced diabetes mellitus model.

Taken together, the present data demonstrate that ExSt treatment in STZ‐induced experimental DM confers hepatic and pancreatic protection and exerts antioxidant and antifibrotic effects, particularly through CAT activation and downregulation of profibrotic cytokines (Figure 8). These results reinforce the therapeutic potential of *S. tuberosa* fruit peel extract for DM management, especially concerning the morphological improvement of chronic complications. However, further studies are required to better elucidate the molecular mechanisms underlying ExSt activity in experimental DM.

## Author Contributions

**Vitória Natália Ferreira de Sena:** conceptualization, methodology, software, investigation, writing – original draft, formal analysis, data curation. **Cecília Paulino Cassiano da Silva:** writing – original draft, methodology, conceptualization, investigation. **Letícia Alves Borges e Pires:** conceptualization, methodology, data curation, formal analysis, software, investigation. **Ludmila Thainá Chaves Freitas:** methodology. **Raimundo Fernandes de Araújo Júnior:** funding acquisition, writing – review and editing, methodology, resources. **Bento João Abreu:** writing – review and editing. **Islania Giselia Albuquerque Araujo:** methodology, writing – review and editing, validation, resources. **Isac Almeida de Medeiros:** methodology, validation, writing – review and editing. **Bruno Raniere Lins de Albuquerque Meireles:** resources, validation, methodology. **Rafael Andre da Silva:** methodology, data curation. **Diego Dias dos Santos:** methodology, data curation, software. **Cristiane Damas Gil:** resources, methodology, data curation, writing – review and editing, funding acquisition. **Karina Carla de Paula Medeiros:** project administration, data curation, supervision, resources, writing – review and editing, methodology, funding acquisition, investigation, conceptualization, formal analysis.

## Funding

This study was financed in part by the Coordenação de Aperfeiçoamento de Pessoal de Nível Superior—Brasil (CAPES)—Finance Code 001.

## Disclosure

All authors read and approved the manuscript.

## Conflicts of Interest

The authors declare no conflicts of interest.

## Data Availability

The data that support the findings of this study are available from the corresponding author upon reasonable request.
